# Neurological manifestations of tuberous sclerosis complex: the importance of early diagnosis

**DOI:** 10.1055/s-0042-1759689

**Published:** 2022-12-19

**Authors:** Anna L. R. Pinto

**Affiliations:** 1 Boston Children's Hospital, Department of Neurology, Division of Epilepsy and Clinical Neurophysiology, Boston, United States.; 2 Harvard Medical School, Boston, United States.

Tuberous sclerosis complex (TSC) is a genetic disorder with an incidence of 1:6000 live births. TSC affects multiple organ systems in widely variable presentations and is characterized by high rates of neurological and neuropsychiatric comorbidities. The genetic diagnosis is possible by identification of pathogenic mutations in either *TSC1* , located on chromosome 9q34 and encoding hamartin, or *TSC2* , located on chromosome 16p13.3 and encoding tuberin. Hamartin and tuberin form a heterodimer that suppresses the mTOR pathway, which coordinates various aspects of cell functioning, including cell growth, differentiation, metabolism and proliferation. 1

The natural history of TSC indicated that symptoms might present differently between pediatric and adult individuals, reinforcing the importance of keeping independent clinical and genetic criteria. According to the International Consensus published in 2021, all individuals suspected of having TSC, regardless of age, should undergo MRI of the brain to assess for the presence of (sub-)cortical tubers and for other neuronal migration defects, subependymal nodules, and subependymal giant cell astrocytomas (SEGAs). 1

Systemic manifestations of the disease, such as cardiac rhabdomyomas, can be detected on routine prenatal ultrasound, and brain involvement can be assessed in prenatal MRI by identification of features described above, allowing definite prenatal diagnosis. 2

Epilepsy is highly prevalent and one of the major risk factors of developmental disorders associated with TSC, such as intellectual disability, autism and TSC-associated neuropsychiatric disorders (TAND). Because the first seizures usually appear during the first year of life, this offers a potential window for pre-symptomatic intervention. Studies have shown that abnormal electroencephalography (EEG) frequently precedes the onset of clinical seizures by several weeks to months. Thus, spikes on routine surveillance EEG are thus a reliable biomarker in TSC for impending onset of epilepsy. The International Consensus recommends routine EEG in asymptomatic infants with TSC every 6 weeks up to age 12 months and every 3 months up to age 24 months. Different types of seizures may coexist in the course of TSC. The most destructive are infantile spasms, which occur in approximately 30–60% of patients. 1

Recent studies have shown a potential beneficial role of preventative antiepileptic treatment in TSC patients, with the possibility of improved outcomes in epilepsy and in cognition. For example, the EPISTOP trial demonstrated a potential neuroprotective effect from pre-symptomatic treatment, but the study was limited by its open label design and small enrollment numbers. The PREVeNT trial is formally studying the preventative use of vigabatrin in a double-blind, placebo controlled multicenter design, and will evaluate both 3-year epilepsy and neurodevelopmental outcomes. Another early intervention is epilepsy surgery, which are performed at an increasingly young age, aiming to prevent developmental fallout from recurrent seizures and medication exposure. 3 4

The rapid development of precision medicine has led to novel needs in terms of evidence generation. Studies describing the natural history of TSC, along with reliable biomarkers and better understanding of the disease pathway, will allow the development of appropriate treatments. For instance, the mTOR inhibitor everolimus has been evaluated in randomized controlled clinical trials specifically to treat seizures in TSC, and was found to be an effective therapeutic option. 5 6

The study published by Pereira et al. 7 in this issue of *Arquivos de Neuro-Psiquiatria* reflects a single tertiary center experience embedded in the Brazilian healthcare system. However, their report demonstrates care that is current and up to date with other major centers across the world. I encourage the interested reader to keep a close eye on TSC, as it is serving as a model disease for rapid advances and even paradigm shifts in the field of pediatric epilepsy.
